# Supplementary material for: Olive Pomace Extract Acts as a New Potent Ferroptosis Inhibitor in Human Cells
Source: Molecules. 2025 Jul 24;30(15):3095. doi: 10.3390/molecules30153095 (PMC12348466; doi:10.3390/molecules30153095)
Supplement: Supplementary file 1 [file molecules-30-03095-s001.zip › molecules-3713653-supplementary.pdf]

Supplementary Material

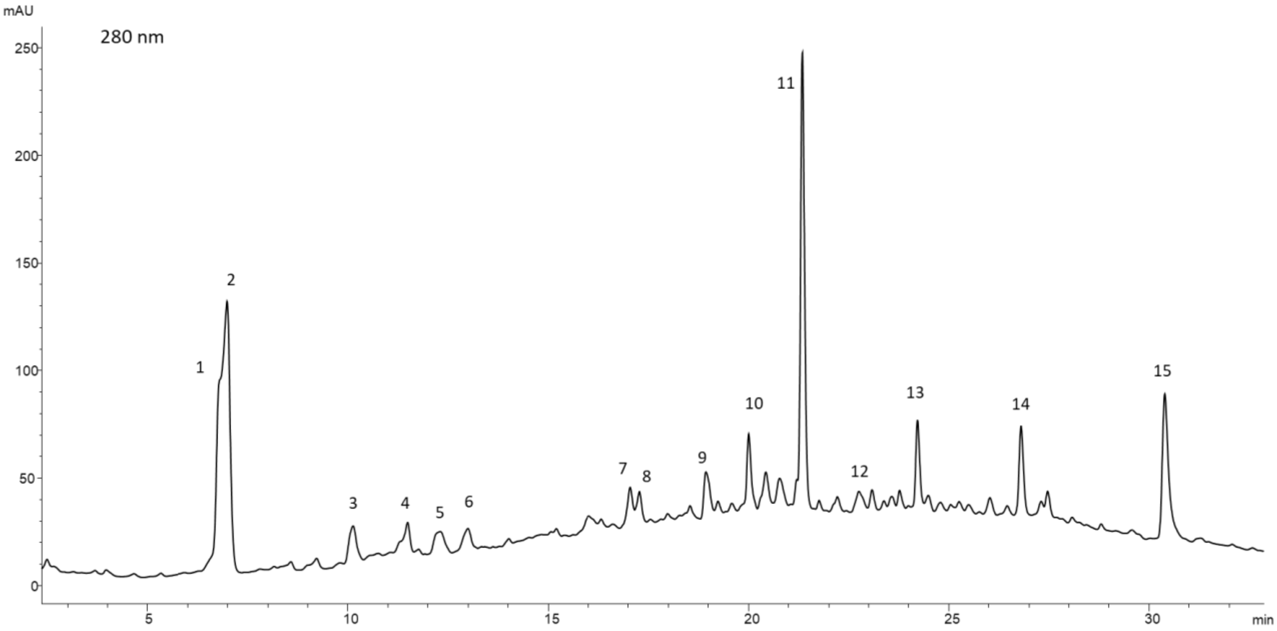

Figure S1.

HPLC chromatogram at 280 nm of OPE. The identity of numbered peaks is provided in table S1.

Table S1. HPLC-quantified phenolic compounds content (mg/g) in lyophilized olive pomace (OP). Data are expressed as mean ± standard deviation (SD) of three independent analyses.

Peak Number	Molecule	Mean	SD
1	OH-tyr hexose	0,71	0,12
2	OH-tyr	1,14	0,11
3	tyr	0,24	0,02
4	tyr derivate	0,13	0,01
5	tyr derivate	0,15	0,02
6	caffeic acid	0,12	0,02
7	OH-acteoside 1	0,13	0,02
8	OH-acteoside 2	0,11	0,02
9	flavonoid	0,08	0,01
10	flavonoid	0,10	0,01
11	verbascoside	1,42	0,15
12	verbascoside iso	0,09	0,00
13	cafselogoside	0,24	0,04
14	comselogoside	0,28	0,03
15	luteolin	0,306	0,030
	*total tyr + OH-tyr	4,96	0,28

* Obtained after acid hydrolysis
